# The impact of computed high b-value images on the diagnostic accuracy of DWI for prostate cancer: A receiver operating characteristics analysis

**DOI:** 10.1038/s41598-018-21523-6

**Published:** 2018-02-21

**Authors:** Peigang Ning, Dapeng Shi, Geoffrey A. Sonn, Shreyas S. Vasanawala, Andreas M. Loening, Pejman Ghanouni, Piotr Obara, Lewis K. Shin, Richard E. Fan, Brian A. Hargreaves, Bruce L. Daniel

**Affiliations:** 1 Zhengzhou University People’s Hospital (Henan Provincial People’s Hospital), Department of Radiology, Zhengzhou, 450003 China; 2 Stanford University School of Medicine, Department of Urology, Stanford, CA94305 USA; 3 Stanford University School of Medicine, Department of Radiology, Stanford, CA94305 USA; 4 VA Palo Alto Health Care System, Department of Radiology, Palo Alto, CA94305 USA

## Abstract

To evaluate the performance of computed high b value diffusion-weighted images (DWI) in prostate cancer detection. 97 consecutive patients who had undergone multiparametric MRI of the prostate followed by biopsy were reviewed. Five radiologists independently scored 138 lesions on native high b-value images (b = 1200 s/mm^2^), apparent diffusion coefficient (ADC) maps, and computed high b-value images (contrast equivalent to b = 2000 s/mm^2^) to compare their diagnostic accuracy. Receiver operating characteristic (ROC) analysis and McNemar’s test were performed to assess the relative performance of computed high b value DWI, native high b-value DWI and ADC maps. No significant difference existed in the area under the curve (AUC) for ROCs comparing B1200 (b = 1200 s/mm^2^) to computed B2000 (c-B2000) in 5 readers. In 4 of 5 readers c-B2000 had significantly increased sensitivity and/or decreased specificity compared to B1200 (McNemar’s p < 0.05), at selected thresholds of interpretation. ADC maps were less accurate than B1200 or c-B2000 for 2 of 5 readers (P < 0.05). This study detected no consistent improvement in overall diagnostic accuracy using c-B2000, compared with B1200 images. Readers detected more cancer with c-B2000 images (increased sensitivity) but also more false positive findings (decreased specificity).

## Introduction

Prostate cancer is the most common solid organ malignancy in American men, accounting for an estimated 220,800 new cases and 27,540 deaths in 2015^1^.

Transrectal ultrasonography (TRUS) remains the imaging modality of choice for prostate cancer diagnosis at biopsy, yet it performs poorly in identification of individual cancer foci. Multiparametric magnetic resonance imaging (mpMRI) clearly exceeds the performance of ultrasound in the detection of prostate cancer. MpMRI has a variety of important clinical applications including predicting reclassification among men who elect active surveillance(AS)^2^ and accurately localizing tumors prior to biopsy^3,4^. Pre-biopsy MRI enables image-targeted biopsy via direct MRI-guidance^5^or MRI-transrectal ultrasound (TRUS) fusion-guided biopsy^6–8^. These methods both improve diagnosis of clinically significant prostate cancer when compared to conventional biopsy methods.

MpMRI of the prostate includes T1- and T2-weighted anatomic imaging and functional MR techniques, including diffusion weighted imaging (DWI), dynamic contrast-enhanced imaging, and MR spectroscopic imaging^9^. DWI is a functional imaging technique that probes tissue structures at the cellular level. It is one of the important components of the Prostate Imaging and Reporting Archiving Data System (PI-RADS). Normal glandular prostate tissue on MRI gives no signal reduction on the ADC map and no increase in signal intensity on the high b-value images (PI-RADS 1). In contrast, aggressive prostate cancer gives a reduced signal on the ADC map (dark) and focal hypersignal intensity on high b-value images (bright) (PI-RADS 4 or 5)^10^. The apparent diffusion coefficient (ADC) value of suspected tumor lesions, a parameter derived from DWI, can often predict not only the presence of prostate cancer but also the cancer grade^11^. ADC values have been shown to be a useful marker for predicting insignificant prostate cancer in candidates for AS^12–14^.

Despite its role as the most important component of mpMRI, DWI is limited by artifacts from physiologic motion, susceptibility, and chemical shift. These are compounded by inherent limitations in signal-to-noise ratio and image resolution. Improving DWI performance is an important goal that would improve the clinical utility of mpMRI.

While it is generally recognized that high b-value images are particularly important in prostate cancer identification, the optimal b-value for tumor detection remains controversial. Although higher b-values offer stronger diffusion-weighting and greater suppression of benign prostate tissue, thus potentially improving tumor conspicuity, these gains may be offset by reduced signal-to-noise ratio as well as increased susceptibility artifact and image distortion^15^. Computed high b-value DWI is a recently described technique in which DW images using very high b-values can be mathematically derived from lower b-value images, rather than directly acquired^16^. In a recent study of 49 patients with 2 radiologists, computed high b-value images increased sensitivity for detection of prostate cancer^17^. The purpose of this paper was to investigate the impact of computed high b-value imaging on image quality and tumor detection, including the impact on both sensitivity and specificity, in a study with multiple radiologists.

## Results

Patient median age was 65 (Interquartile range = 4). Biopsy histopathology was available from targeted biopsy of all 138 lesions in the 97 patients. 77 lesions were benign and 61 were malignant on biopsy. The Gleason score of the malignant lesions ranged from Gleason 6 to Gleason 10. The most common Gleason score was 3 + 4 (25 lesions). Details of the biopsy indication, prostate-specific antigen (PSA) level, lesions per patient, lesion size and lesion location are shown in Tables 1 and 2.

**Table 1:** Study population characteristics.

	Range	Average	Number(n)
Age (years)	45–79	64 ± 6	40–59(n = 23)
			60–69(n = 56)
			70–79(n = 18)
PSA (ng/mL)	0.6–63	10.7 ± 8.0	<4(n = 4)
			4–10(n = 55)
			>10(n = 38)
Lesions per patient	1–5	1.4 ± 0.8	1 lesion(n = 70)
			2 lesions(n = 18)
			3 lesions(n = 6)
			4 lesions(n = 1)
			5 lesions(n = 2)
Indication			No Prior Biopsy; Elevated PSA(n = 56)
			Negative Prior Biopsy; Rising PSA(n = 37)
			Active Surveillance for known Prostate Cancer(n = 42)
			Staging for clinically significant Prostate Cancer(n = 1)
			Other(n = 1)

n: patient number; PSA: Prostate-Specific Antigen.

**Table 2:** Lesion characteristics.

	Range	Average	Number(n)
Lesion size(mm)	5–40	13.3 ± 6.4	5–9(n = 46)
			10–19(n = 73)
			20–40(n = 19)
Lesion location in gland			L(n = 73); R(n = 65)
			PZ (n = 63); TZ(n = 7); AFS(n = 5); ML(n = 69); SV(n = 2)
			Apex(n = 32); Mid(n = 97); Base(n = 22);
Gleason score			G 6 (n = 15); G 7 (n = 33); G 8 (n = 8); G 9(n = 4);G 10 (n = 1)

n: lesion number; L: left; R: right; PZ: peripheral zone; TZ: transition zone; AFS: anterior fibromuscular stroma; ML: median lobe; SV: seminal vesicle; G 6: Gleason 3 + 3; G 7: Gleason 3 + 4 and Gleason 4 + 3; G 8: Gleason 3 + 5 and Gleason 4 + 4; G 9: Gleason 4 + 5 and Gleason 5 + 4; G 10: Gleason 5 + 5.

**Figure 1:**
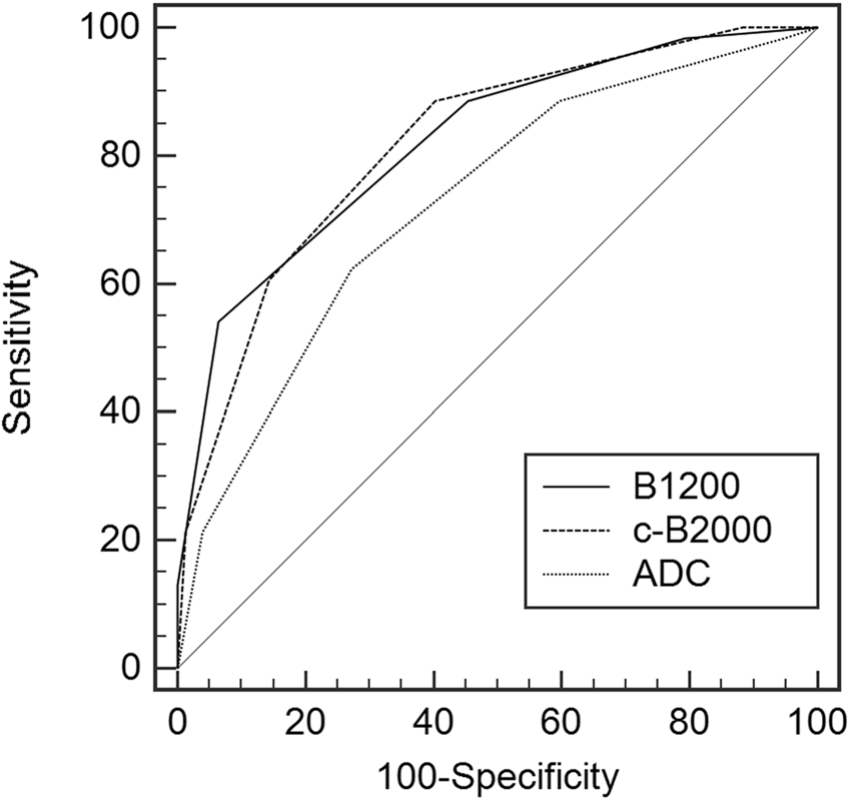
ROC Comparison of Diagnostic Accuracy of DWI method for Reader 1. The ROC curves for B1200 and c-B2000 are nearly identical.

The AUCs and asymptotic 95% confidence interval for differentiating benign from malignant lesions using B1200, c-B2000 or ADC for each reader are shown in Table 3. AUC comparisons are shown in Table 4. No significant difference existed between the B1200 and c-B2000 AUCs for any of the 5 readers, as indicated by the complete overlap of corresponding AUC values for the B1200 images with the 95% confidence intervals for the c-B2000 images, and vice-versa, for every reader. For example, the ROC comparison of diagnostic accuracy using different DWI methods for Reader 1 in Fig. 1 showed nearly identical ROC curves for B1200 and c-B2000. While no differences were identified in diagnostic accuracy between B1200 and c-B2000, differences were seen between high b-value DWI and ADC for some readers. The AUC of B1200 exceeded that of ADC in 2 of 5 readers (p < 0.05). The AUC of c-B2000 exceeded ADC in 3 of 5 readers (p < 0.05) as shown in Table 4.

**Table 3:** AUCs of B1200, c-B2000 and ADC for Benign vs Tumor.

	B1200	c-B2000	ADC	95% Confidence Interval		
				B1200	c-B2000	ADC
Reader 1	0.821	0.818	0.726	[0.751,0.891]	[0.748,0.888]	[0.641,0.811]
Reader 2	0.832	0.798	0.717	[0.763,0.901]	[0.742,0.872]	[0.632,0.803]
Reader 3	0.842	0.824	0.823	[0.771,0.913]	[0.750,0.897]	[0.748,0.898]
Reader 4	0.859	0.904	0.807	[0.796,0.921]	[0.850,0.958]	[0.732,0.882]
Reader 5	0.846	0.815	0.807	[0.778,0.913]	[0.742,0.889]	[0.732,0.882]
Average	0.840	0.832	0.776			

B1200: b value = 1200 s/mm^2^, c-B2000:computed b value = 2000 s/mm^2^, ADC: apparent diffusion coefficient.

**Table 4:** The AUC Comparison of B1200, c-B2000 and ADC for Benign vs Tumor.

	P value		
	B1200-c-B2000	B1200-ADC	c-B2000-ADC
Reader 1	0.926	0.014*	0.009*
Reader 2	0.308	0.005*	0.014*
Reader 3	0.626	0.655	0.984
Reader 4	0.136	0.206	0.002*
Reader 5	0.230	0.353	0.824

Asterisks indicate significance at p ≤ 0.05.

We also looked for significant differences in diagnostic accuracy between readers by comparing the AUC of their B1200 ROC curves. The AUC values for each reader fell in a narrow range from 0.821 to 0.859. No significant differences were found between the readers; AUC values for each reader fell squarely within the 95% confidence intervals of the other readers.

**Figure 2:**
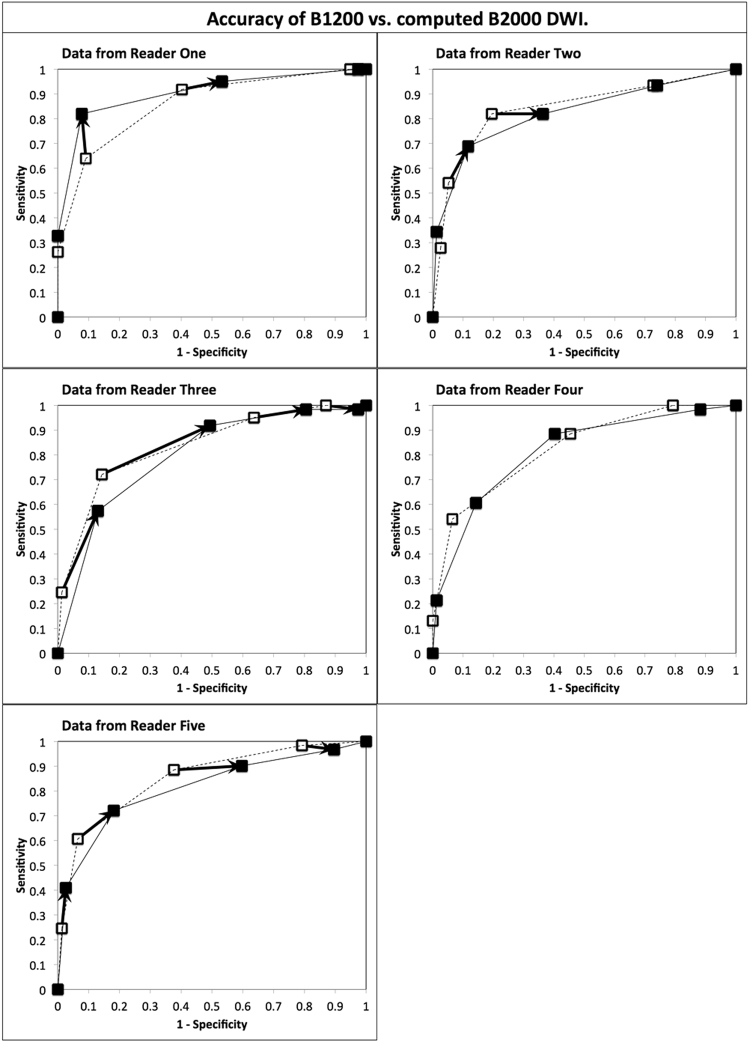
Receiver operating characteristic curve analysis comparing the diagnostic accuracy of direct B1200 diffusion-weighted prostate MR images with computed B2000 images in five readers. Open squares with dashed lines are from direct B1200 data. Solid squares with solid lines are from computed B2000 images. Arrows connect corresponding B1200 and cB2000 operating points when there is a difference in sensitivity and/or specificity that is significant by two-tailed McNemar’s test for comparisons of paired proportions, at a level of p < 0.05. Overall 12 operating points demonstrated different sensitivity and or specificity in 4 of 5 readers. In reader 4 there were no significant differences between corresponding pairs of operating points at B1200 and computed B2000. The ROC curve areas and overall ROC curve shapes remained the same for all readers, indicating that despite changes in sensitivity and specificity for some readers at some thresholds of interpretation, overall computed B2000 images do not increase intrinsic diagnostic accuracy compared to direct B1200 images.

Given that previous authors have reported increases in sensitivity with computed high b-values, the sensitivity and specificity values of B1200 and c-B2000 were compared at different thresholds of interpretation provided by the 5-point rating scale. The four “operating-points” or interpretation thresholds were 1 vs. 2–5, 1–2 vs. 3–5, 1–3 vs. 4–5 and 1–4 vs. 5. At each of these thresholds, sensitivity and specificity of each method were calculated and compared for B1200 and c-B2000 using McNemar’s test. These results are shown in Fig. 2 for all four operating points for all 5 readers (for a total of 20 points). In 4 of 5 readers there were significant increases in sensitivity and/or decreases in specificity with c-B2000 compare to B1200 (McNemar’s p < 0.05) at selected thresholds of interpretation (Fig. 2). As an example, the differences in sensitivity and specificity of B1200 vs. c-B2000 at the interpretation threshold of 1 or 2 vs. 3–5 are given in Table 5. Overall the average reader sensitivity for prostate cancer at this threshold increased from 61% to 75% while the corresponding specificity decreased from 92% to 80%. Detailed data for other interpretation thresholds are not shown.

**Table 5:** Comparison of Diagnostic Accuracy when a Rating 3, 4 or 5.

	Sensitivity			Specificity		
	B1200	c-B2000	p-value	B1200	c-B2000	p-value
Reader 1	0.639	0.820	0.008*	0.909	0.922	0.705
Reader 2	0.541	0.689	0.039*	0.948	0.883	0.025*
Reader 3	0.721	0.918	0.001*	0.857	0.506	0.000*
Reader 4	0.541	0.607	0.346	0.935	0.857	0.083
Reader 5	0.607	0.721	0.052*	0.935	0.818	0.007*
Reader Average	0.610	0.751		0.917	0.797	

(McNemar’s 2-tailed p-value. Asterisks indicate significance at p ≤ 0.05).

We identified 11 false-positive lesions where two or more readers scored the lesion as suspicious (score of 4 or 5) on computed B2000 images but scored it as benign (score of 1, 2, or 3) on direct B1200 images. In these 11 cases, pathology reports were unrevealing, and described the histologic pattern as “benign prostatic glands and stroma” in all 11 lesions. In one of these 11 lesions, one of 4 cores also showed some equivocal atypical small acinar cell proliferation but this one incidental finding was not enough to explain the false positives in our study. Of note, 9/11 of these lesions were located in the transition zone, including all five lesions where three or more readers scored a false positive.

Data were also analyzed to investigate whether c-B2000 images were more diagnostic of higher grade tumors than B1200 images. For this analysis, lesions were classified as “non-high grade lesions” (benign and Gleason 3 + 3) versus “high-grade tumors” (Gleason 3 + 4, or higher). There were no significant differences in ROC AUCs between B1200 and c-B2000 for any of the readers (data not shown).

## Discussion

The main results of this study are that computed very high b-value images (c-B2000) increase the sensitivity with which radiologists detect prostate cancer on DWI compared to native high b-value images (B1200). This study reports twice as many patients as the previous largest study^17^ and includes an ROC analysis of results from 5 radiologists. But, the c-B2000 images also reduce the specificity with which radiologists exclude benign findings.

Diffusion-weighted MRI is a key part of both the first and second PI-RADS standards for interpretation of prostate MRI^10,18^. Numerous studies have shown that restricted diffusion is the single most reliable predictor of cancer on multi-parametric MRI. Some recent studies have proposed that imaging with very high b-values (b = 1500 mm^2^/sec or greater) may be most accurate^19^. In practice, FDA regulations, scanner hardware performance, and imaging artifacts limit the achievable gradient slew rates, and peak gradient amplitude, which in turn lead to tradeoffs in very-high b-value DWI protocols. Standard DWI imaging with very high b-values suffers from longer echo times, increase T2-weighting, increased distortions, “ghosting” artifacts, and increased spatial blurring due to eddy currents during the echo-planar readout. Very high b-value DWI also requires substantially longer scan time in order to obtain enough signal averages to compensate for the intrinsic reduction in signal-to-noise ratio that occurs with direct high b-value imaging.

**Figure 3:**
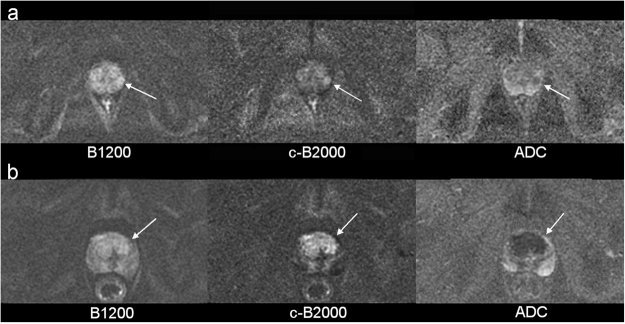
Examples of cases where Computed B-value increased the conspicuity of prostate cancer. (**a**) A small (8 mm) Gleason 4 + 4 tumor in the left latera peripheral zone of a 54 y/o man is faintly seen on B1200 images (left) but is more visible as a dominant abnormality compared to background variation in signal on computed B2000 images (center). (**b**) Simlarly, a large (40 mm) bilateral Gleason 3 + 3 tumor in the transition zones of a 72 y/o man is also more consipicuous on computed B2000 images (center).

Recently, computed diffusion-weighted images have been proposed as an alternative method to achieve images with similar contrast as native high b-value DWI, by extrapolating from images obtained with lower b-values^20,21^. Computed B1500 images improve the contrast between the high signal of prostate cancer and the normal background signal in the peripheral zone^16,17^. This increase in conspicuity of cancers translated to increased detection of prostate cancer; in a two-reader study comparing computed B1500 images with direct B1000 images in n = 49 men who underwent radical prostatectomy, Rosenkrantz *et al*.^17^ found that computed B1500 images increased the sensitivity for prostate cancer from 46.9% to 69.4% and from 46.9% to 67.3% for reader 1 and 2, respectively. Similarly, Grant *et al*.^22^ reported that cancer detection increased from 57/90 (63%) on direct B1000 images to 79/90 (88%) on computed B2000 images, using a multi exponential IVIM signal model. Bittencourt^23^ also detected 3 more cancers with computed high b-value images in a cohort of 24 patients. The results of our study agree with these previous studies. For example, as detailed in Table 5 in our study, using a reader rating of “slightly, clearly or markedly hyperintense to overall gland signal” (corresponding to a rating of 3, 4 or 5) as indicative of a positive test result, the average sensitivity for prostate cancer increased from 61% on direct B1200 images to 75% on c-B2000 images for five readers (p < 0.05 for 4 of 5 readers). Together the results of this study and the previous studies indicate that prostate cancer detection can be increased by post-processing to compute very high b-value images (Fig. 3).

**Figure 4:**
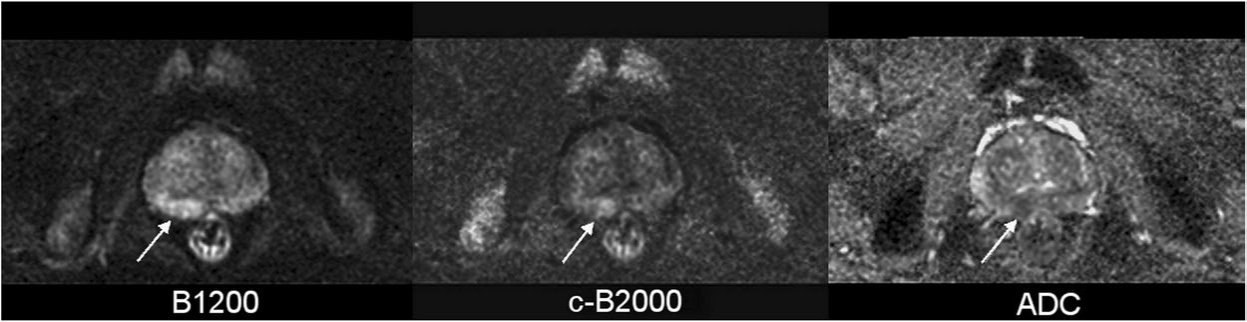
Example of false positive Computed B-2000 image. A small focal high signal area (10 mm) of the medial right peripheral zone of a 51 y/o man was most conspicuous on c-B2000 images (center arrow). TRUS-fusion biopsy revealed only benign prostate glandular tissue, confirming that this finding was a false-positive abnormality on c-B2000.

Increased cancer detection does not necessarily imply an overall increase in diagnostic accuracy. It is important to determine whether c-B2000 images improve overall discrimination of benign and malignant findings, or whether they increase detection of *both* true positives *and* false (Fig. 4). The largest previous studies reporting increased detection did not report on the impact of computed high b-value images on specificity^17^. Table 2 in the paper by Grant *et al*. suggests that false positives increased from 75/149 (50%) on direct B1000 images to 95/149 (64%) on c-B2000 images^22^. In contrast, one small study of 10 patients found that sensitivity and specificity both increased from 89.4% and 87.5% to 96.0% and 96.6%, respectively with computed high b-value images^16^. Due to the uncertainty about the impact of cB2000 on specificity, our study was performed using a 5-point reader-rating scale, thereby enabling full ROC analysis of diagnostic accuracy of direct B1200 and computed B2000 images. The very similar shapes and lack of increased areas under the ROC curves for direct B1200 and computed B2000 images for all 5 readers are consistent with the data from Grant *et al*.^22^ that computed high b-value images increase detection of both true and false positives, and indicate that computed high b-value images do not increase overall diagnostic accuracy. Most of the false positives on computed B2000 images were merely due to the overall lower repeatability of MRI in the transition zone, a well known phenomenon noted in numerous previous studies.

The results of the ROC analysis also confirm a somewhat higher diagnostic accuracy for B1200 as well as c-B2000 images compared to ADC map images, as supported by the higher ROC AUCs for c-B2000 images (average AUC 0.832) compared to ADC maps (average AUC 0.776) that was observed for all five readers (Table 3), and statistically significant for three of them (Table 4). This supports PI-RADS scoring schemes that recommend inspection of high b-value images, rather than merely reviewing ADC maps^10,18^.

This study has several unique features compared to previous work. The DWI images were performed with a novel pulse sequence for reduced field-of-view diffusion MRI that provides high quality images with relatively little distortion and blurring^24^ and were performed without use of an endorectal coil. Direct comparison with other DWI pulse sequences, including full field-of-view accelerated DWI, readout-segmented echo-planar imaging (EPI)^25^ and images performed with an endorectal coil was not possible in this retrospective study.

The study had several limitations. The retrospective design also limits the study. Although consecutive patients were included without regard to clinical indication, tumor size, etc., some selection bias is possible because B1200 images and ADC maps may have contributed to the original radiologist’s decision to select the lesions for MRI-TRUS fusion biopsy, but computed high b-value images did not. In addition, MRI-TRUS fusion biopsy was used as the gold standard for pathologic diagnosis because imaging targeting avoids potential mis-registration/mis-assignment errors that may occur between prostatectomy specimens and pre-operative images. However, it is possible that some cancers were missed which could impact the results, particularly for small lesions. Also, 24/121 (20%) of patients with available images and pathology were excluded because of unacceptable image quality. Finally, the very high b-value images were calculated using a mono exponential model^16,17,23^ but were not compared to other more complicated multi-exponential signal models incorporating Kurtosis or intravoxel incoherent motion (IVIM)^22^.

In summary, this study extends previous reports that computed high-b value images increase the sensitivity of clinical DWI for prostate cancer using a clinically relevant DWI protocol that does not require directly acquiring B2000 images in a larger number of patients interpreted independently by multiple radiologists, with image-directed biopsy as the gold standard. However, this increased sensitivity is accompanied by a commensurate increase in false positives. Rather than raising overall increase diagnostic accuracy, computed high b-value images are best described as moving radiologists to a more sensitive but less specific operating point on their DWI ROC curve.

## Methods

### Study design

**Figure 5:**
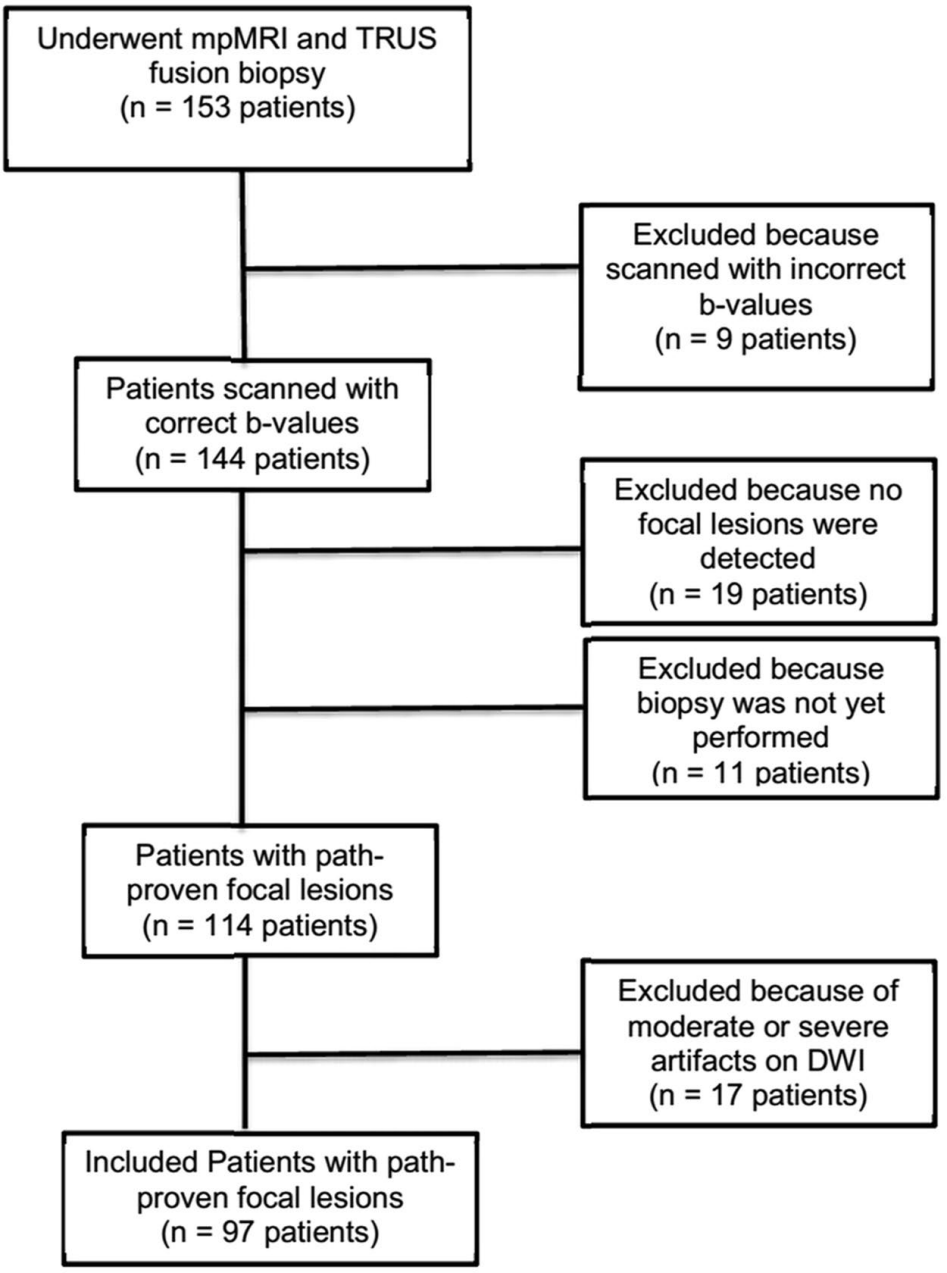
Flow chart of patients included and excluded.

This retrospective study was approved by Stanford’s institutional review board with a waiver of the requirement for written informed consent. We identified 153 consecutive patients who underwent mpMRI of the prostate followed by MRI–TRUS fusion biopsy. Our clinical practice was to use PI-RADS 1 and all lesions classified as PI-RADS 3, 4 or 5 during the initial clinical review of scans were targeted for biopsy. Pathology was only from MRI-TRUS fusion biopsies. Our MRI-TRUS fusion biopsies were performed at target locations on images. Those locations were used to direct radiologists where to provide their interpretations. We directed the radiologists to the lesion with a “line” on the images but not a contour or circle so as to avoid pre-biasing the radiologists about the potential extent of disease at any particular target location. All subjects with adequate DW imaging, available pathology, and multiple b-values including b = 50, 800 and 1200 s/mm^2^ were included. We excluded 56 subjects due to incorrect b-values on DWI or unavailable pathology result or moderate or severe artifact. In total, 97 patients with 138 MRI lesions were included (Fig. 5).

### DWI

Diffusion weighted imaging of the prostate was performed using a 3 Tesla MR scanner (MR750, GE Healthcare, Waukesha, WI) and using an external 32 channel body array coil in prostate mode, without an endo-rectal coil. A dedicated small field-of-view spin-echo echo-planar sequence used a 2-dimensional radio-frequency pulse to selectively excite spins in a narrow rectangular strip of tissue, enabling higher resolution prostate images with a shorter echo-planar read-out train. This achieves high resolution images of the prostate with minimal distortion^24,26^. A slice-selective refocusing pulse only refocuses on-resonant water spins, avoiding fat spins because they are not excited in the same slice. B-values of 0, 50, 800, 1200 sec/mm^2^ were used in all patients, using tetrahedral encoding. Number of signal averages was 12, 24, and 64 for b-values 50, 800, and 1200 respectively. Other scan parameters included TR/TE of 2000/54 ms, Field of view of 24 cm (frequency encoding direction) × 12 cm (phase encoding direction), Corresponding matrix (160 × 80), Slice thickness was 4.0–4.2 mm, Fourteen slices were obtained through the prostate and seminal vesicles.

### Imaging processing

DWI images were analyzed using open-source DICOM software Osirix (version 6.5 64-bit, Pixmeo, Geneva, Switzerland), with the ADC plug-in “ADCmap” to calculate the ADC maps from the b = 50, 800 and 1200 s/mm^2^ source images by fitting the signal to a mono-exponential decay function: I (b) = I (0)exp(−b*D) where I is the signal intensity, b is the b-value, and D is the diffusivity on a pixel-by-pixel basis. The same function was used with a b = 2000 s/mm^2^ to calculate the computed b2000 (c-B2000) images.

Overall, this protocol and imaging processing yielded three types of diffusion-dependent images which were used in the reader study: Native EPI-DWI with b = 1200 sec/mm^2^ (B1200), computed DWI at b = 2000 sec/mm^2^ (c-B2000), and maps of the apparent diffusion coefficient (ADC maps).

### Reader study

**Figure 6:**
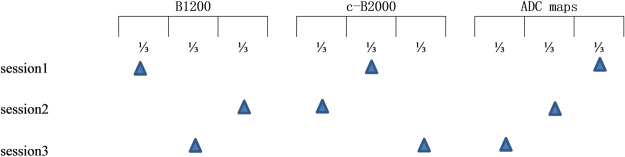
Flow chart of the reader study.

In order to maximize the generalizability of this study, five board-certified radiologists analyzed the images independently. These readers had 1, 2, 4, 4, and 7 years of experience reading prostate MRI. And they were body-MRI fellowship trained attendings (4) or board-eligible body-MRI fellowship trainee (1). The readers only interpreted the designated spots where biopsy was performed. The radiologists reviewed the images in 3 sessions, separated by at least 3 days, to minimize learning bias. During each session, each radiologist reviewed the B1200 images on one third of the lesions, c-B2000 images on the second third of the lesions, and ADC maps on the final third of the lesions. During the second session the radiologists reviewed c-B2000 images on the first third of the lesions, ADC maps on the second third of the lesions and B1200 images on the final third of the images. During the third session, the radiologists reviewed the ADC maps on the first third of the lesions, the B1200 images on the second third of the lesions, and the c-B2000 images on the final third of the images (Fig. 6). In this fashion, during each session, each radiologist reviewed every lesion once, with one of the three types of images. After completing all three sessions, each radiologist had reviewed every lesion 3 times, once with each type of image. This design was chosen to minimize the influence of the results from one type of images on a radiologists’ interpretation of the other types of images (learning bias), and to allow the diagnostic accuracy of each type of image to be independently assessed. A 5-point scoring system was used to score the images (shown in Table 6). While similar to PI-RADS version 1^18^, this scale was used instead of the PI-RADS because it allows each method to be analyzed independently rather than integrating the interpretation of ADC and high b-value DWI images. We did not strictly follow PI-RADS v1 or PI-RAD v2 for the reader analysis because we wanted to specifically look at the diagnostic accuracy contribution of each type of images separately; the PI-RADS do not assign separate scores to the ADC and DWI images.

**Table 6:** The 5-point scoring system for B1200, c-B2000 and ADC.

score	B1200 and c-B2000	ADC
1	Normal. No increase in signal intensity (SI)	No lesion
2	Focal, slightly hyper intense, but similar to overall heterogeneity	Barely visible, similar to overall heterogeneity
3	Focal, dominant, slightly hyper intense compared to overall heterogeneity	Focal, slightly decreased ADC compared to overall heterogeneity
4	Focal, dominant, clearly hyper intense compared to overall heterogeneity	Dominant, Focal, Moderately decreased ADC compared to overall heterogeneity
5	Focal, dominant, markedly hyper intense with no competing heterogeneity	Dominant Focal, markedly low ADC, without significant heterogeneity elsewhere

MedCalc Statistical Software (version 14.8.1; MedCalc Software bvba, Ostend, Belgium; http://www.medcalc.org; 2014) was used for statistical analysis. Receiver operating characteristic (ROC) analysis and McNemar’s test of paired proportions was performed to assess the sensitivities and specificities of the B1200 images, c-B2000 images and ADC maps with distinguishing benign from malignant. Area under the ROC curve (AUC) was compared.
